# Retrospective Evaluation of Metformin and/or Metformin Plus a New Polysaccharide Complex in Treating Severe Hyperinsulinism and Insulin Resistance in Obese Children and Adolescents with Metabolic Syndrome

**DOI:** 10.3390/nu9050524

**Published:** 2017-05-20

**Authors:** Stefano Stagi, Franco Ricci, Martina Bianconi, Maria Amina Sammarco, Giovanna Municchi, Sonia Toni, Lorenzo Lenzi, Alberto Verrotti, Maurizio de Martino

**Affiliations:** 1 Department of Health Sciences, University of Florence, Anna Meyer Children’s University Hospital, Florence 50139, Italy; Frank8622@hotmail.it (F.R.); martinabianconi88@gmail.com (M.B.); mary.sammarco@hotmail.com (M.A.S.); maurizio.demartino@unifi.it (M.d.M.); 2 Department of Paediatrics, University of Siena, Siena 53100, Italy; g.municchi@technet.it; 3 Paediatric Diabetology Unit, Anna Meyer Children's University Hospital, University of Florence, Florence 50139, Italy; s.toni@meyer.it (S.T.); lorenzo.lenzi@meyer.it (L.L.); 4 Department of Paediatrics, University of L’Aquila, L’Aquila 67100, Italy; alberto.verrottidipianella@univaq.it

**Keywords:** children, adolescents, metformin, Policaptil Gel Retard^®^, obesity, type 2 diabetes mellitus, haemoglobin A1c, treatment, hyperinsulinism, insulin resistance, metabolic syndrome, insulin, cholesterol, triglycerides

## Abstract

**Background:** Pharmacological treatment of obesity and glucose-insulin metabolism disorders in children may be more difficult than in adults. Thus, we evaluate the effects of metformin in comparison with metformin plus a polysaccharide complex (Policaptil Gel Retard^®^, PGR) on body weight and metabolic parameters in obese children and adolescents with metabolic syndrome (MetS). **Patients and methods**: We retrospectively collected 129 children and adolescents (67 girls, 62 boys; median age 12.6 years) treated for a minimum of two years with metformin and low glycemic index (LGI) diet. Of these, 71 patients were treated with metformin plus PGR after at least 12 months of metformin alone. To minimize the confounding effect of the LGI on auxological and metabolic parameters, the patients were compared with age-, sex-, and BMI-matched control group with obesity and MetS (51 subjects; 24 males, 27 females) treated only with a LGI diet. Assessments included lipids, glucose and insulin (fasting and after oral glucose tolerance test) concentrations. The Homeostatic Model Assessment of Insulin Resistance (HOMA-IR), Matsuda, insulinogenic and disposition indices were calculated. **Results:** Metformin treatment led to a significant reduction in BMI SDS (*p* < 0.0001), with a significant difference in ΔBMI SDS between patients and controls (*p* < 0.0001). Moreover, metformin treated patients showed a reduction in HOMA-IR (*p* < 0.0001), HbA1c levels (*p* < 0.0001) and a significant increase in Matsuda index (*p* < 0.0001) in respect to the reduction discovered in controls (*p* < 0.05). Moreover, in contrast to the group treated with metformin alone and controls, patients treated with metformin plus PGR showed a further reduction in BMI SDS (*p* < 0.0001), HOMA-IR (*p* < 0.0001), HbA1c (*p* < 0.0001), total, HDL and LDL cholesterol (*p* < 0.0001), as well as an increase in Matsuda (*p* < 0.0001), disposition (*p* < 0.005) and insulinogenic (respectively, *p* < 0.05 and *p* < 0.0001) indices. **Conclusions:** Metformin appears to show short-term efficacy in reducing BMI, adiposity and glucose and insulin parameters in obese children and adolescents with MetS. However, PGR added to metformin may be useful to potentiate weight loss and to improve glucose-insulin metabolism and adiposity parameters in these patients.

## 1. Introduction

Obesity is a multifactorial disease with a rapidly increasing prevalence in children and adolescents and a significant impact on both physical and psychosocial health [1]. More than one third of children and adolescents are reportedly at risk of being overweight or obese in Italy [2] and many other European countries [3,4,5].

Concomitant with the global rise in pediatric obesity, there has been a significant increase in the number of children and adolescents with clinical signs of insulin resistance [6,7], the main pathophysiological event preceding metabolic syndrome abnormalities [6,7,8,9]. Furthermore, the incidence of impaired fasting glucose, impaired glucose tolerance and type 2 diabetes (T2DM) in obese children and adolescents has risen alarmingly [6,7,8,9]. 

Lifestyle changes such as a healthy diet and regular physical activity have been proposed as the gold standard of care in subjects with obesity, albeit with poor compliance and success [6,7,8,9]. However, given that insulin resistance is an important link between obesity and associated metabolic abnormalities and cardiovascular risk, clinicians should be aware of high risk groups and the necessary treatment approaches [6,7].

There is now a general consensus that the pharmacological treatment of T2DM or severe disorders involving insulin secretion and action may be more difficult in children than in adults [10]. The first problem is that most of the available medications have not been studied in children [10]. 

The American Diabetes Association and the Pediatric Endocrine Society recently prepared joint guidelines for the treatment of T2DM in children [11,12]. Metformin, a biguanide agent that decreases hepatic glucose production and increases peripheral insulin sensitivity, has been used in conjunction with a lifestyle intervention program in T2DM obese adolescents with clinical insulin resistance to achieve weight loss and improve insulin sensitivity [13]. As in adults, metformin remains the mainstay of therapy (alongside diet and exercise) in T2DM children and adolescents, even if the treatment should focus on lifestyle changes to achieve effective weight management [10]. 

In any case, metformin may be a useful adjuvant treatment in obese and/or insulin-resistant children and adolescents, although its role in this setting is still unclear [14,15,16]. 

Nevertheless, although lifestyle changes may produce significant weight reduction in children and adolescents, the long-term efficacy of lifestyle intervention programs on body mass index (BMI) and related complications is questionable, given the high dropout and the frequent relapse into obesity of these patients [17]. For this reason, adding a pharmacological agent to conventional treatment is often considered in clinical practice.

Metformin must be taken with food. This can be a problem in children and adolescents, who often find it difficult to manage their eating, potentially leading to hypoglycemia [18]. Metformin may also be associated with gastrointestinal adverse events, commonly including abdominal pain and diarrhea [19]. Although rare, lactic acidosis may also occur [20]. 

Policaptil Gel Retard^®^ (PGR) is a complex of polysaccharide macromolecules that may reduce peak blood glucose and insulin levels [21]. It may thus be able to reduce BMI, HbA1c levels, the frequency of acanthosis nigricans and glucose metabolism abnormalities in obese children and adolescents with severe hyperinsulinism, insulin resistance and a family history of T2DM and obesity [21]. 

The aim of this study was to retrospectively evaluate the effect of metformin and/or metformin plus PGR in treating severe hyperinsulinism and insulin resistance in a large cohort of obese children and adolescents with metabolic syndrome (MetS).

## 2. Patients and Methods

This was a retrospective single center study. One hundred twenty-nine Caucasian obese patients (67 females, 62 males; median age at study entry 12.6, range 8.1–14.3 years) with hyperinsulinism and insulin resistance associated with MetS were studied at the Paediatric Endocrinology Unit at the Meyer Children’s University Hospital of Florence, Italy. 

Inclusion criteria were the presence of severe hyperinsulinism, insulin resistance and MetS and age between 8.0 and 14.5 years at the first evaluation. Exclusion criteria were patients aged <8.0 years or >14.5 years at the first evaluation, cognitive impairment, diagnosis of type 1 diabetes, existing syndrome disorders with or without cognitive impairment, impaired renal or hepatic function, malabsorption disorders, cancer, patients enrolled in a weight loss program at the first evaluation, endocrine causes of obesity such as hypothyroidism or Cushing disease, and use of medications for weight loss or any medication that could compromise the study evaluation such as topical or systemic glucocorticoids, anticonvulsant therapy, growth hormone, sexual steroids or gonadotropin releasing hormone analogues.

This study was conducted in compliance with the Declaration of Helsinki and European Guidelines on Good Clinical Practice. Ethical approval (ethical code 122/2016) was obtained from the Meyer Children’s University Hospital Ethics Committee. Written informed assent/consent was obtained from all participants and their parents or guardians. 

## 3. Study Design

One hundred twenty-nine patients recruited for severe obesity, hyperinsulinism and insulin resistance associated with MetS were retrospectively evaluated. After the first evaluation, all patients started metformin (T0) and they were followed up after 12 months (T1; median age 13.7, range 9.1–15.4 years) and 24 months (T2; median age at study end 14.9, range 10.3–16.7 years). Because of data demonstrating improvements in adiposity and gluco-insulinemic parameters with the use of PGR in obese children and adolescents with family history of obesity and T2DM [21], we after the initial 12 months of metformin treatment we recruited 71 patients also treated with PGR (Group A), while 58 patients continued with metformin only (Group B). These patients were compared with a age-, sex-, and BMI-matched control group with obesity and MetS (51 subjects; 24 males, 27 females; median age at study entry 12.4, range 8.2–14.5 years) not taking a medication as a mean to reduce weight and treated only with a low glycemic index (LGI) diet. 

At the first evaluation (T0), the parents of the study participants also completed a previously tested health questionnaire and a semi-structured quality-of-life questionnaire [19,20]. Information on duration of pregnancy, birth weight, neonatal feeding and presence of diabetes gravidarum during the pregnancy of the participants’ mothers and on diseases, hospital admissions and use of medication was collected. Family history was also investigated, collecting data on hypertension, obesity, hypercholesterolemia, cardiovascular disease (myocardial infarction, stroke, transient ischemic attack, and peripheral arterial occlusions) and diabetes mellitus in first degree (parents) and second degree (grandparents, brothers, sisters) family members. Complementary information was also collected from the medical files. Nutrient diaries were logged for all subjects according to their medical charts and through standardized interviews [19,20]. 

At T0, T1 and T2, we collected, when available, clinical and demographic data including height, weight, BMI, waist (WC) and hip circumference (HC), waist–hip ratio (WHR), pubertal staging and the time dedicated to outdoor physical activity, using a questionnaire commonly administered at the medical evaluation in obese children in our hospital [21]. 

During the T0, T1 and T2 visits, an extensive physical examination was performed by the study physicians. This included auscultation of the heart, lungs and abdomen and abdominal palpation. Any abnormal findings were recorded. The skin was examined for acanthosis nigricans, striae rubra, acne and, in girls, hirsutism. 

At T0 blood was taken for glycosylated hemoglobin (HbA1c), oral glucose tolerance test (OGTT), renal profile, lipid profile (including total, HDL LDL cholesterol and triglycerides), full blood count, IgA anti-tissue transglutaminase antibody (tTG) and total IgA levels. HbA1c, OGTT, renal profile, lipid profile and full blood count were also repeated at T1 and T2.

Finally, in addition to the data obtained from telephone interviews, a questionnaire was completed at T1 and T2 to assess any side effects that had occurred during the study. However, as per clinical practice, all patients underwent a telephone interview every three months to monitor BMI changes and any adverse events of treatments. 

As for clinical practice in our Unit, because of research demonstrating improvements in adiposity and gluco-insulinemic parameters [21], all patients with obesity and MetS were treated with a LGI diet. Subjects were visited or interviewed every three months by a dietician (M.A.S.), for nine sessions during the 24-month intervention period, to receive standardized instructions for healthy eating and exercising. Counseling sessions included the child and at least one parent, when possible, according the established practice. Each session lasted approximately 30 min and was based on a strategy of increasing energy expenditure and modifying the dietary food intake using lifestyle behavioral change to achieve long-lasting impact. The dietician completed a tracking form and progress note after each counseling session to document patterns of dietary intake. Regarding the physical exercise, we encourage children to do aerobic exercise, such as swimming, cycling, running, and dancing, 2–3 times per week. 

### 3.1. Metformin Treatment

After the T0 evaluation results, in the daily practice, subject’s study medication dose was progressively increased according to a prespecified algorithm: in Weeks 1 and 2, participants took one tablet (500 mg) daily; thereafter, the dosage was increased by 500 mg/day every seven days to a maximum dose of 1500 mg/day (three tablets). Treatment was administered during meals (to minimize gastrointestinal side effects and the risk of hypoglycemia). In the event that the participant developed gastrointestinal symptoms, the dosage was reduced to the last well-tolerated dosage. For example, we decreased the dose by 250 mg/dose for one week when participants reported difficulty tolerating study medication and then attempted to increase it. 

### 3.2. Policaptil Gel Retard^®^ (PGR) Treatment

As previously reported [21], PGR is the active pharmaceutical ingredient (API) of the medical device Libramed tablets (Aboca Spa Company, Sansepolcro, Arezzo, Italy). This complex contains polysaccharide macromolecules (cellulose, hemicellulose, pectin, and mucilage) and is derived from the following high-fiber raw materials: glucomannan (*Amorphophallus konjac*), cellulose, Opuntia pulp stem (*Opuntia ficus indica*), chicory root (*Cichorium intybus*), freeze-dried mallow root mucilage (*Althaea officinalis*), freeze-dried flaxseed mucilage (*Linum usitatissimum* L) and freeze-dried linden flower mucilage (*Tilia platyphyllos Scop*). PGR slows the rate of carbohydrate absorption, hence potentially reducing peak blood glucose and insulin concentrations. The exact composition and production process of the API are covered by a European patent (no. 1679009). All patients took three tablets (2175 mg) before their two main meals.

### 3.3. Adherence to Study Protocol

Adherence to therapy was evaluated by means of written instructions provided at T0 and at clinical controls through a written questionnaire completed by the parents. As per clinical practice, adherence was also verified by e-mails and telephone interviews and by the bottle count on number of tablets consumed performed at the programmed visits. Furthermore, adherence to the diet was measured through the food record and 24-h recall of all food and drink intake and was revealed on the individual consultation, when specific questioned were asked about the food record.

### 3.4. Control Group

Patients with obesity and MetS, who chose not to take a medication as a mean to reduce weight and treated only with a LGI served as a control group (51 subjects; 24 males, 27 females; median age at study entry 12.4, range 8.2–14.5 years). For minimize the confounding effect of the LGI on auxological and metabolic parameters, this control group was age-, sex-, and BMI-matched and had no statistical different auxological and metabolic parameters at T0 in respect to the study group. Inclusion and exclusion criteria and the study protocol of the control group were the same as previously seen for patients.

### 3.5. Study Protocol

As reported above, at T0, T1 and T2, the habitual food intake of the patients in the six months prior to and during the study was assessed using a validated quantitative food frequency questionnaire [21,22]. Standardized pictures of small, medium and large food portions were used to increase the accuracy of estimated food consumption [21]. 

The glycemic index (GI) of the diet of each patient was estimated from the sum of the GI values of each food consumed daily (GIA, GIB, GIC, etc. according to the amount (in grams) of available carbohydrate in each food (gA, gB, gC, etc.) divided by the total amount of available carbohydrate (g), as described by Wolever et al. [23]. The GI of each food was obtained from the values published in the International Table of Glycemic Index, considering glucose as reference [24]. For foods not listed in this table, the GI of foods with a similar nutritional composition and preparation method was used. GI values of the edible products were assumed as following: GI < 55 low, GI = 56–70 medium, GI > 70 high [23]. 

As per clinical practice, the study’s dietician provided to ask a complete a three-day food record (including two weekdays and two weekend days) at baseline and again three months. These four-day food diaries provided information on dietary composition and served as the basis of individualized dietary counseling. A 24-h recall of all food and drink intake was conducted during each session to assess compliance. In the case of noncompliance, suitable alternative LGI foods were encouraged. Food sample baskets containing key foods for the assigned diet were provided to promote product recognition and dietary adherence. LGI have a target GI ≤ 55. Our eating guideline encouraged the addition of fiber (exchanging white bread to whole meal bread) and legumes (lentil, peas, and beans), and focused on designing meals using low-GI carbohydrate foods. Examples of LGI foods used in this study: legumes (beans, chick pea, and lentils), whole grains (oat, barley, bulgur wheat, cracked wheat, semolina, basmati rice, and all bran), temperate fruits (apples, berries, pears, apricot, peach, plums, etc.), citrus fruits (oranges, grapefruit, tangerine, and pineapple), “above the ground” vegetables (squash, mixed vegetables, green beans, broccoli, tomato juice, tomato sauce, vegetable soups, asparagus, cauliflower, spinach, cabbage and onions, leafy greens, all above ground growing vegetables, and carrots), breads (pumpernickel and whole grain), and cereals (muesli with whole grain flakes, raw bran, etc).

The age of pubertal onset was defined as the age at durable Tanner B2 stage for females or a testicular volume of 14 mL for males (G2). The age at which this occurred was taken as mid-age between the previous clinic visit when the child was still prepubertal and the clinic visit when the child was G2/B2. Duration of puberty was taken as time from G2/B2 to G4/B4. Age at G4/B4 was assessed similarly by taking mid-age between the previous clinic visit when the child was G3/B3 and the clinic visit when the child was G4/B4 [25].

Obesity was defined according to the reference values in growth charts as shown in the study by Cacciari et al. [26]. Children with a BMI greater than the 95th percentile for their age and gender were classified as obese. 

The variables for insulin resistance and β-cell function were evaluated in all patients by OGTT, carried out at T0, T1 and T2. OGTT was performed at four time points after an overnight fast of 12 h. After insertion of a venous cannula and collection of the baseline blood sample (T0), participants ingested 1.75 g of glucose per kilogram of body weight (maximum dose 75 g), dissolved in 200–300 mL of water. Glucose and insulin levels were determined at baseline and 30, 60, 90 and 120 min after ingestion of the glucose solution [22]. 

The glycemic status was defined based on 2010 American Diabetes Association criteria [27]. Impaired fasting glucose (IFG) was defined as fasting plasma glucose (FPG) 100–125 mg/dL (5.6–6.9 mmol/L). Impaired glucose tolerance (IGT) was defined as OGTT 2-h value 140–199 mg/dL (7.8–11.0 mmol/L). Finally, diabetes was defined as FPG ≥ 126 mg/dL (≥ 7.0 mmol/L) and OGTT 2-h plasma glucose ≥ 200 mg/dL (≥ 11.1 mmol/L).

The HOMA-IR and the Matsuda index of insulin sensitivity [28,29,30] were calculated for all patients. A low HOMA-IR index indicates high insulin sensitivity, whereas a high HOMA-IR index indicates low insulin sensitivity (insulin resistance). HOMA-IR > 4.4 was considered as consistent with insulin resistance [30,31]. The Matsuda index [29] also provides a measure of insulin sensitivity and is calculated using the following equation:

Matsuda index = 10,000/(square-root (FPG x FPI) × (meanPG x meanPI)

Where FPG is fasting plasma glucose, FPI is fasting plasma insulin, PG is plasma glucose and PI is plasma insulin. The Matsuda index is consistent with direct measurements using an insulin clamp [29].

The glucose and insulin area under the curve (AUC) during the OGTT were calculated using the trapezoidal rule [32]. Delta glucose (ΔG30–0) and delta insulin (ΔI30–0) were evaluated as the changes in glucose and insulin concentrations from 0 min to 30 min. The insulinogenic index, calculated as (Ins30-Ins0)/(Glu30-Glu0), was used to estimate insulin secretion [33]. The β-cell compensatory capacity was evaluated using the disposition index (DI), defined as the product of the Matsuda and insulinogenic indices [33].

MetS was diagnosed applying the International Diabetes Federation (IDF) 2007 definition according to different age groups: 6 to <10, 10 to <16, and ≥16 years. For children aged 10 to <16 years, MetS was diagnosed by the presence of abdominal obesity (WC ≥ 90th percentile for age and gender) plus two or more of the other features: elevated TG (≥1.7 mmol/L), low HDL-C (<1.03 mmol/L), high blood pressure (BP) (systolic (SBP) ≥ 130 mmHg or diastolic (DBP) ≥ 85 mmHg) and elevated blood glucose (≥5.6 mmol/L) [34,35,36]. IDF criteria for adults [36] were used to identify MetS in those aged ≥16 years. For children under 10 years, the individual risk components of MetS were defined as for children aged 10 to <16 years [36].

### 3.6. Auxological and Clinical Methods

Height was measured, in triplicate, to the nearest 0.1 cm using a wall-mounted Harpenden stadiometer in the Anna Meyer Children’s Hospital. All measurements were carried out by the same trained staff members. The coefficient of variation (CV) for these measurements was <1%. Weight was recorded on a digital scale to the nearest 0.05 kg, with the subject shoeless and dressed in light underwear. BMI was calculated as body weight divided by height squared (kg/m^2^). Height, weight and BMI were stratified using Italian growth charts [26]. Pubertal staging was carried out according to Tanner and Whitehouse’s criteria, using an orchidometer for the boys [37].

Waist circumference was measured to the nearest 0.1 cm at the end of normal expiration using a non-elastic tape measure placed midway between the lowest rib margin and the iliac crest [36,37]. Hip circumference was measured to the nearest 0.1 cm using a tape measure positioned horizontally over the widest part of the gluteal region as the subject stood relaxed with his/her feet placed as close together as possible [38,39]. All measurements were taken twice. The waist/hip ratio was calculated using these measurements [40]. 

Height, weight, BMI, waist circumference and hip circumference were normalized for chronological age by conversion to standard deviation scores (SDSs) [25]. 

Acanthosis nigricans on the neck was assessed for severity by a validated scale ranging from grade 0 (not present) to grade 4 (severe: extending anteriorly, visible when the participant is viewed from the front) [41].

Blood pressure was measured three times by trained personnel by auscultation using a mercury sphygmomanometer on the right arm after the patient had been sitting quietly for 5 min, with the back supported, feet on the floor, right arm supported and cubital fossa at heart level [42]. An appropriate cuff size was used and the 5th Korotkoff sound was taken for diastolic blood pressure categorization. The mean systolic and diastolic values were recorded and stratified according to the pediatric percentiles of the National High Blood Pressure Education Program Working Group on High Blood Pressure in Children and Adolescents [42]. The mean value of the three measurements was also converted into SDS to enable some of the statistical analyses [42].

### 3.7. Laboratory Methods

All participants were examined in the morning after an overnight fast. Blood samples were collected by venepuncture or, for OGTT, by venous cannula, during scheduled hospital visits. After collection, serum and plasma were immediately separated and stored at −20 °C in multiple vials for later analysis. All samples were collected by research staff and analyzed in the clinical laboratory of the Anna Meyer Children’s University Hospital.

Serum glucose (Dimension RXL system, Dade Behring, Dallas, TX, USA) and serum insulin (IMMULITE 2000 analyzer, Siemens Healthcare Diagnostics, Marburg, Germany) levels were measured using immunoenzymatic assays and glycosylated haemoglobin (HbA1c) levels were determined using high performance liquid chromatography (DIAMAT, Bio-Rad, Richmond, CA, USA). The normal range for HbA1c was 4.2%–6.0%, and the coefficient of variation (CV) at 5.5% was 4.8.

Total cholesterol, HDL cholesterol, and triglyceride (TG) were measured using routine laboratory methods. Low-density lipoprotein (LDL) cholesterol was calculated using the Friedwald formula: LDL  =  total cholesterol − HDL cholesterol − TG/2.2 [30].

## 4. Statistical Analysis 

Data were analyzed using IBM SPSS Statistics software version 22 (IBM, Armonk, NY, USA). Baseline data are reported as descriptive statistics. Normally distributed data are reported as mean ± SD, and nonparametric data as median (range). To assess the effect of PGR plus metformin versus metformin alone, Student’s T test was used to compare the means of normally distributed data and the Mann–Whitney U test to compare nonparametric data. The *χ*2 test or Fisher's exact test, when appropriate, was used for dichotomous outcomes (progression of impaired fasting glucose, impaired glucose tolerance and T2DM). General linear models (analysis of repeated measure (analysis of variance or ANOVA)) or mixed models (in cases of too many missing data) were used to determine the effects of each type of treatment on different time points to baseline. Tukey honest significance difference test was used for post hoc multiple comparisons. Differences with *p* < 0.05 were considered significant.

## 5. Results

The primary demographic, clinical and laboratory characteristics of patients and controls are summarized in Table 1 and Table 2. All baseline characteristics were similar for both males and females (Table 1), and there were not significantly more girls than boys in puberty (Tanner stage 2–5). A family history of MetS in either first or second degree relatives was found for 99 patients (76.7%), without significant difference with the controls (41 patients, 80.4%). 

### 5.1. Metformin Intervention

At the onset of metformin treatment (T0) 66 patients were prepubertal and 63 were pubertal (28 in Tanner stage 2 and 35 in stages 3–5), without significant difference in respect to the controls (27 prepubertal, 10 in Tanner 2 and 14 in stages 3–5). 

Regarding SDSs, we did not discover significant differences between patients and controls (Table 1). Furthermore, both patients and controls showed a significant imbalance of energy intake with a high-protein, high-fat, high-sugar carbohydrate and poor-fiber diet than expected in Italian non-obese peers [43]. However, at T0 we not discovered significant difference in relation to the mean GI, energy intake, consumption of fiber, fat, carbohydrate and protein in patients and controls (Table 1). 

Seventy-six patients (58.9%) presented acanthosis nigricans (controls: 27 patients, 52.9%). All patients and controls had MetS: waist SDS was > 2 in all patients and controls; 49/129 (38.0%) patients and 16/51 (31.4%) controls presented impaired fasting glucose; 53/129 (41.1%) patients and 25/51 (49.0%) controls presented elevated TG, 113/129 (87.6%) patients and 41/51 (80.4%) controls presented reduced HDL cholesterol levels, 68/129 (52.7%) patients and 29/51 (56.9%) controls presented elevated SBP and 56/129 (43.4%) patients and 27/51 (47.1%) controls presented elevated DBP. 

At T0 glucose-insulinemic metabolism and metabolic parameters did not show statistically significant differences between patients and controls: HOMA-IR 7.42 ± 1.94 (controls: 6.99 ± 1.81), Matsuda index 1.22 ± 0.29 (controls: 1.31 ± 0.27), disposition index 4.73 ± 3.84 (controls: 4.12 ± 3.71), insulinogenic index 3.78 ± 2.81 (controls: 3.63 ± 2.70), HbA1c 6.29 ± 0.31% (controls: 6.19 ± 0.32%), total cholesterol 5.82 ± 0.61 mmol/L (controls: 5,74 ± 0.73 mmol/L), HDL cholesterol 0.81 ± 0.14 mmol/L (controls: 0.83 ± 0.13 mmol/L), LDL cholesterol 4.20 ± 0.65 mmol/L (controls: 4.12 ± 0.59 mmol/L), triglycerides 1.77 ± 0.31 mmol/L (controls: 1.93 ± 0.40 mmol/L), ALT 57.23 ± 20.89 U/L (controls: 53.67 ± 18.91 U/L), AST 59.66 ± 27.53 U/L (controls: 55.21 ± 24.90), SBP SDS 1.53 ± 0.92 (controls: 1.56 ± 0.99), DBS SDS 1.56 ± 0.78 (controls: 1.61 ± 0.84) (Table 1).

As expected, we did show statistically significant differences between prepubertal and pubertal subjects both in patients and controls regarding HOMA-IR, Matsuda index, disposition index, insulinogenic index and HbA1c levels. However, the comparison of prepubertal patients and controls groups and pubertal patients and controls groups did not allow showing significant differences between the groups relatively to the examined parameters (data not shown).

After 12 months of metformin treatment (T1), there was no significant difference in height SDS in patients (0.50 ± 0.93 vs. 0.55 ± 0.96) and controls (0.56 ± 0.88 vs. 0.53 ± 0.91), whereas there was a significant reduction in BMI SDS in patients (2.18 ± 0.21 vs. 2.44 ± 0.25, *p* < 0.0001) and controls (2.31 ± 0.24 vs. 2.42 ± 0.26, *p* < 0.05), with a significant difference in ΔBMI SDS between patients and controls (−0.26 ± 0.04 vs. −0.11 ± 0.02, *p* < 0.0001), revealing that metformin has a significant effect on weight reduction (Table 1). Moreover, patients treated with metformin disclosed significant differences regarding to waist SDS (2.78 ± 0.52 vs. 3.14 ± 0.63; *p* < 0.0001), hip SDS (3.73 ± 0.65 vs. 4.08 ± 0.72; *p* < 0.0001) and WHR (0.88 ± 0.05 vs. 0.87 ± 0.02; *p* < 0.05). On the contrary, patients treated with LGI no showed a significant reduction in waist SDS (2.99 ± 0.61 vs. 3.12 ± 0.66), hip SDS (4.01 ± 0.71 vs. 4.10 ± 0.70) and WHR SDS (0.88 ± 0.05 vs. 0.89 ± 0.06). Furthermore, patients and controls had significant differences with regard to Δwaist SDS (−0.36 ± 0.11 vs. 0.13 ± 0.05; *p* < 0.0001), Δhip SDS (−0.35 ± 0.07 vs. −0.09 ± 0.01; *p* < 0.0001), but no ΔWHR SDS (−0.01 ± 0.03 vs. −0.01 ± 0.01). These differences were not significative comparins prepubertal and pubertal patients and controls.

Twenty-one patients (41.1%) presented acanthosis nigricans, a significant reduction from T0 (*p* < 0.0005), with significant difference in respect to the control group (54.9%; *p* < 0.005). Furthermore, there was also a significant reduction in patients in HOMA-IR (6.11 ± 1.23 vs. 7.42 ± 1.94; *p* < 0.0001) and insulinogenic index (2.95 ± 1.96 vs. 3.78 ± 2.81; *p* < 0.05), and a significant increase in Matsuda index (1.51 ± 0.22 vs. 1.22 ± 0.29; *p* < 0.0001). There was no significant difference in disposition index from T0 (4.53 ± 3.38 vs. 4.73 ± 3.84). On the contrary, controls showed a significant reduction in HOMA-IR (6.23 ± 1.94 vs. 6.99 ± 1.81; *p* < 0.05) and a significant increase in Matsuda index (1.42 ± 0.24 vs. 1.31 ± 0.27; *p* < 0.05), whereas insulinogenic index (3.51 ± 2.04 vs. 3.63 ± 2.70) and disposition index (4.43 ± 3.74 vs. 4.12 ± 3.71) did not showed significant differences. Finally, HbA1c was significantly reduced in patients (6.01 ± 0.35 vs. 6.29 ± 0.31; *p* < 0.0001) and controls (6.03 ± 0.28% vs. 6.19 ± 0.32%; *p* < 0.05), whereas HDL cholesterol (0.88 ± 0.14 vs. 0.81 ± 0.14 mmol/L; *p* < 0.0001) was significantly increased and LDL cholesterol (3.98 ± 0.68 vs. 4.20 ± 0.65 mmol/L; *p* < 0.05) decreased in patients.

Nevertheless, both in patients than in controls, there were no significant differences in total cholesterol, triglycerides, ALT, AST, SBP SDS or DBS SDS (Table 1). Finally, all patients still presented MetS: waist SDS > 2 in all patients; impaired fasting glucose in 28/129 (21.7% vs. 38.0% at T0; *p* < 0.0001); elevated TG in 68/129 (52.7% vs. 41.1% at T0; *p* < 0.05), reduced HDL cholesterol in 111/129 (86.0% vs. 87.6% at T0; *p* = NS), elevated SBP in 59/129 (45.7% vs. 52.7% at T0; *p* = NS) and elevated DBP in 41/129 (31.7% vs. 43.4% at T0; *p* < 0.005). On the contrary, in controls impaired fasting glucose was discovered in 29.4% (vs. 31.4% at T0; *p* = NS), elevated TG in 68.6% (vs. 80.4% at T0; *p* < 0.05), reduced HDL cholesterol in 76.5% (vs. 80.4% at T0; *p* = NS), elevated SBP in 52.9% (vs. 56.9% at T0; *p* = NS) and elevated DBP in 43.1% (vs. 47.1% at T0; *p* < 0.005). 

However, after 12 months of metformin treatment, we did show statistically significant differences between prepubertal and pubertal patients in respect to respective subjects of the control group and between prepubertal and pubertal subjects of the control group, but not between prepubertal and pubertal patients, relatively to HOMA-IR, Matsuda index, disposition index, insulinogenic index and HbA1c levels (data not shown). 

The result of the dietary analysis after the 6-week of LGI diet showed a slight change in caloric intake both in patients (2008 ± 364 Kcal vs. 2,206 ± 458 Kcal; *p* < 0.005) and in controls (1975 ± 386 vs. 2,181 ± 443 kcal; *p* < 0.05), without significant differences between the two groups. Furthermore, at T1, the mean GI was also significantly reduced in patients (47.6 ± 7.3 vs. 59.3 ± 15.5; *p* < 0.0001) and controls (47.7 ± 7.2 vs. 58.9 ± 16.2; *p* < 0.0001). Furthermore, there were favorable not significant changes in some macronutrient intakes: protein intake increased (patients: 89.8 ± 20.7 vs. 94.6 ± 19.9 g; controls: 91.4 ± 23.1 vs. 96.5 ± 22.3) and carbohydrate intake decreased (Patients: 286.0 ± 75.9 g vs. 299 ± 64.4 g; controls: 286.5 ± 70.4 g vs. 297.0 ± 69.1 g), even if with significant differences between complex and simple carbohydrates, as demonstrated by the significant reduction in the glycemic index. However, fat intake significantly decreased (patients: 62.8 ± 20.3 vs 77.7 ± 20.2 g, *p* < 0.0001; controls: 61.7 ± 23.4 g vs. 74.9 ± 19.7 g, *p* < 0.0001) and fiber intake increased (patients: 20.8 ± 9.4 g vs. 16.5 ± 7.8 g, *p* < 0.0001; controls: 21.5 ± 9.6 vs. 16.0 ± 7.2 g, *p* < 0.005) (Table 1).

### 5.2. Metformin Plus Policaptil Gel^®^ Retard Versus Metformin Alone

At T1, after dividing the patients into groups A (metformin plus PGR) and B (metformin only), there were no significant differences in metformin treatment doses between the group A and B and between prepubertal and pubertal patients (data not shown), physical activity scores (data not shown), LGI diet characteristics (Table 2). Furthermore, we did not disclosed significant differences in male/female ratio and prepubertal/pubertal ratio among the two groups of patients and the controls (Table 2). Moreover, there were no significant differences in height SDS waist SDS, WHR, HOMA-IR, insulinogenic index, disposition index, HbA1c, total cholesterol, LDL cholesterol, triglycerides, ALT, AST, SBP SDS or DBP SDS among the two groups of patients and the controls, whereas patients showed significant differences in BMI SDS (*F*: 3.890; *p* < 0.05), hip SDS (*F*: 3.391; *p* < 0.05), Matsuda index (*F*: 3.271; *p* < 0.05), and HDL-cholesterol (*F*: 3.720; *p* < 0.05) among the two groups of patients and the controls (Table 2).

At T2, the mean GI was 46.9 ± 7.0 for the for the group A, 47.4 ± 6.8 for the group B and 48.3 ± 6.6 for the controls, without significant differences among the groups, as well as regarding energy intakes, consumed fiber, fat, carbohydrate or protein intake (Table 2). Moreover, also the result of the dietary analysis at T2 showed no change in GI, caloric intake and macronutrient intake in respect to T1 both in patients and in controls (Table 2). 

The comparison at T2 among the three groups disclosed significant differences regarding BMI SDS (*F*: 46.496; *p* < 0.0001), waist SDS (*F*: 20.232; *p* < 0.0001) and hip SDS (*F*: 10.325; *p* < 0.0001). In particular, group A of patients showed BMI SDS and waist SDS values significantly reduced in comparison to group B and the controls (*p* < 0.0001), whereas hip SDS value was significantly reduced in respect to the controls only (*p* < 0.001). Moreover, group B also disclosed reduced values of BMI SDS (*p* < 0.005), waist SDS (*p* < 0.05) and hip SDS (*p* < 0.05) in respect to the controls (Table 2).

In comparison to T1, at T2 Group A (PGR plus metformin) presented a significant reduction in BMI SDS (1.92 ± 0.17 vs. 2.22 ± 0.20; *p* < 0.0001), waist SDS (2.42 ± 0.43 vs. 2.75 ± 0.58; *p* < 0.0005) and hip SDS (3.30 ± 0.41 vs. 3.78 ± 0.69; *p* < 0.0001), whereas in Group B (metformin alone) and controls there was no further statistically significant differences with respect to T1.

Group A and B showed also a significant reduction of patients with acanthosis nigricans (respectively, *p* < 0.0001 and *p* < 0.005) in comparison to controls at T2; however, in group A this reduction was significantly different also in respect to group B (*p* < 0.0001), and in comparison to T1 (*p* < 0.0001) (Table 2). 

The T2 comparison among the groups A and B and the controls disclosed significant differences regarding HOMA-IR (*F*: 56.160; *p* < 0.0001), Matsuda (*F*: 56.059; *p* < 0.0001), insulinogenic (*F*: 9.211; *p* < 0.0001) and disposition (*F*: 9.090; *p* < 0.0001) indexes. In particular, group A of patients showed significant differences in HOMA-IR (*p* < 0.0001), Matsuda index (*p* < 0.0001), disposition index (*p* < 0.005) and insulinogenic index (respectively, *p* < 0.05 and *p* < 0.0001) in respect to the group B and controls. 

Moreover, in comparison to T1, at T2 in both Groups A and B there was a significant increase in insulin sensitivity (A: 2.27 ± 0.52 vs. 1.47 ± 0.19; *p* < 0.0001; B: 1.65 ± 0.21 vs. 1.53 ± 0.25; *p* < 0.05). In Group A there was also a significant reduction in HOMA-IR (4.12 ± 0.47 vs. 6.31 ± 1.31, *p* < 0.0001) and a significant increase in disposition (6.78 ± 2.99 vs. 4.27 ± 2.81, *p* < 0.0001) and insulinogenic (2.24 ± 1.12 vs. 2.81 ± 1.69, *p* < 0.05) indices. However, Matsuda index was also significantly increased in group B in respect to T1 (*p* < 0.05). In contrast, there was no further significant difference in these parameters in the controls. 

At T2, the comparison among the groups A and B and the controls disclosed significant differences in HbA1c levels (*F*: 22.712; *p* < 0.0001), total cholesterol (*F*: 5.060; *p* = 0.0007), HDL cholesterol (*F*: 34.352; *p* < 0.0001), LDL cholesterol (*F*: 15.697; *p* < 0.0001), ALT (*F*: 14.434; *p* < 0.0001) and AST (*F*: 7.498; *p* = 0.0001). In particular, group A disclosed significant reduced HbA1c levels (*p* < 0.0001), total cholesterol (respectively, *p* < 0.005 and *p* < 0.05), HDL cholesterol (*p* < 0.0001), LDL cholesterol (*p* < 0.0001) and ALT (respectively, *p* < 0.05 and *p* < 0.0001) in comparison to group B and controls. Moreover group A showed a significant reduction also of AST in respect to controls (*p* < 0.005). However, group B disclosed a significant increase in HDL cholesterol than controls (*p* < 0.05).

In comparison with Group B, Group A showed a further reduction in HbA1c levels (A: T2 5.71 ± 0.28 vs. T1 5.98 ± 0.41; *p* < 0.0001; B: T2 5.94 ± 0.26 vs. T1 6.04 ± 0.29). Group A also showed a further reduction in total cholesterol (T2 5.18 ± 0.68 vs. T1 5.80 ± 0.58 mmol/L; *p* < 0.0001), LDL cholesterol (T2 3.25 ± 0.90 vs. T1 4.09 ± 0.63 mmol/L; *p* < 0.0001) and triglycerides (T2 1.58 ± 0.15 vs. T1 1.78 ± 0.27 mmol/L; *p* < 0.0001) and an increase in HDL cholesterol (T2 1.06 ± 0.11 vs. T1 0.91 ± 0.14 mmol/L; *p* < 0.0001) (Table 2). In contrast, Group B presented a significant further reduction in triglycerides alone (T2 1.57 ± 0.15 vs. T1 1.88 ± 0.37 mmol/L; *p* < 0.0001). In contrast, there was no further significant difference in these parameters in the controls. 

At T2 group A showed a significant reduction of glucose metabolism abnormalities: patients with IFG were significantly reduced in comparison to controls (group A: *p* < 0.0001; group B: *p* < 0.005) and significantly reduced in comparison to T1 in group A (*p* < 0.0001). However, at T2 patients with IGT or T2DM were significantly reduced, respectively, in group A (*p* < 0.0001) and groups A and B (respectively, *p* < 0.0001 and *p* < 0.005) in comparison to controls, even if only group A disclosed a significant reduction in respect to T1 (respectively, *p* < 0.05 and *p* < 0.005) (Table 2).

Finally, Group A showed a significant reduction in MetS in comparison with Group B (56% vs. 20%; *p* < 0.0001) and controls (group A: 56% vs. 5.8%; *p* < 0.0001. Group B: 20% vs. 5.8%; *p* = 0.005). 

### 5.3. Safety Data and Adherence to Therapy

During the 12 months of metformin treatment (T0–T1), the incidence of adverse events was 20.1% (27 patients); in eight patients the dosage was reduced to 500 mg twice daily, resulting in a significant reduction in symptoms. These events included hypoglycemia (3 patients; 2.3%) diarrhea (8; 6.2%), constipation (3; 2.3%), flatulence (6; 4.6%) and abdominal pain (9; 7.0%). No patient needed to stop the metformin treatment and none suffered from severe gastrointestinal adverse events. 

After division into treatment subgroups (from T1 to T2), metformin was well tolerated by most patients in both Groups A (metformin + PGR) and B (metformin monotherapy). In Group A, twelve patients (16.9%) reported adverse effects such as hypoglycemia episodes (2 patients; 2.8%), diarrhea (4; 5.6%), flatulence (2; 2.8%) and abdominal pain (4; 5.6%). Notably, three of these had also reported symptoms in the T0–T1 period. In Group B, ten patients (17.2%) continued to report moderate symptoms such as diarrhea (3; 5.2%), flatulence (2; 3.4%) and abdominal pain (5; 8.6%). 

Finally, based on pills or tablets counts, written instructions and questionnaires, as well as e-mails and telephone interviews, adherence to metformin therapy was 89% (range 77%–98%), without significantly difference in group A and B. However, the adherence to PGR treatment was 91% (range 83%–99%). 

## 6. Discussion

Our data show that metformin significantly reduces BMI SDS, waist circumference SDS and hip circumference SDS in the short term, revealing a meaningful effect in children and adolescents with obesity. It also seems to improve glucose-insulin metabolism in these patients, significantly reducing HOMA-IR and HbA1c levels and increasing insulin sensitivity as evaluated by the Matsuda index. 

This confirms previous studies in which metformin produced modest but favorable short term effects on body weight, body composition and glucose homeostasis in obese children both with and without insulin resistance [44,45,46,47,48,49,50]. Other authors have reported that metformin also provides a statistically significant (albeit very modest) reduction in BMI when combined with lifestyle changes in the short term [51]. While results from long term follow-up are very rare [52,53], they seem to show a reduction in the effects of metformin on metabolic and adiposity parameters [53], strengthening our results.

However, our data from this long-term retrospective study with 24 months of follow-up show that, after an initial improvement, metformin alone produces no further statistically significant improvements in auxological and metabolic parameters. Treatment with metformin alone thus may not be sufficient to resolve metabolic syndrome in many obese children and adolescents. 

Given the prevalence of metabolic syndrome and diabetes in young adults who were obese as children or adolescents, the exploration of conventional and pharmacological strategies to improve insulin sensitivity is imperative. Efforts to preserve β-cell function before significant loss occurs thus appear to be necessary in the treatment of obese youths with metabolic syndrome, possibly through alternative treatment programs combining metformin with other products so as to maintain good results in the long term. 

Our data suggest that PGR may significantly potentiate and extend the metabolic effects of metformin in obese children and adolescents with severe insulin resistance and metabolic syndrome. This may be of great interest in relation to the rise in comorbidities of childhood obesity, that necessitate new therapeutic approaches to reduce or prevent these complications, particularly in relation to glucose-insulin metabolism. In fact, progression to IGT in adults is characterized by insulin resistance and the inability of the β-cells to adequately compensate for high blood glucose levels through increased insulin secretion [54]. This progression is probably also seen in children and adolescents [55]. However, although the transition from normoglycemia to IGT and subsequently to diabetes in adults is usually a gradual phenomenon that occurs over 5–10 years, the early onset of T2DM in the young suggests an accelerated process with shortening of the transition time between these steps [56].

However, we cannot exclude that the effect shown by metformin or by the association of metformin plus PGR in patients with obesity and MetS may be, at least in part, due to other mechanisms, such as changes in the gut microbiota. Interestingly, metformin treatment may improved the glycemic profile modifying gut microbiota, and accumulating evidence suggests that the gut microbiota is an important factor in mediating the development of obesity-related metabolic disorders, including type 2 diabetes [57,58]. For example, high-fat diet (HFD)-fed C57BL/6 mice treated with metformin showed a higher abundance of the mucin-degrading bacterium Akkermansia muciniphila with an increase of the number of mucin-producing goblet cells due to metformin treatment [59,60,61]. Other studies in animal models disclosed an increase of Clostridium cocleatum [60], Butyrivibrio, Bifidobacterium bifidum, and Megasphaera [61]. Similar data are hypothesized in human with T2DM [62]. Moreover, we have no data on the possible effects of PGR on the gut microbiota. However, future studies in this field will be needed to better understand the metabolic effects of metformin, or products such as PGR in patients with obesity, impaired glucose metabolism and MetS. However, there is increasing interest in utilizing dietary fibers or carbohydrate polymers in the modulation of gut microbiota [63].

Moreover, we previously showed that the PGR complex is useful in improving BMI, metabolic parameters and glucose-insulin metabolism in obese children and adolescents with severe hyperinsulinism and insulin resistance [21]. The present study confirms these results and shows that PGR may be a useful natural tool, in combination with metformin, to achieve an additional reduction in BMI, glucose-insulin and adiposity parameters, reducing the number of patients with MetS to a significantly greater extent than metformin alone. These results highlight interesting new potential effects of this complex. 

Further studies are needed to better evaluate the pharmacokinetics of this association, in particular studying the effect of metformin with PGR from the beginning of the treatment, for better evaluate if its metabolic effect may be more pronounced and stable later. The effect of PGR seems to be related to a reduction in post-meal blood glucose and insulin peaks due to slower glucose absorption, thus attenuating pancreatic insulin response. This may potentiate the effect of metformin and achieve a further decrease in hepatic glucose production, fasting plasma glucose, serum triacylglycerol and VLDL and LDL levels as well as reduce C reactive protein [64]. 

The main limitation of this study is its open-label, retrospective design. This may cause a bias, as participants receiving long-term metformin treatment alone, i.e. those remaining insulin resistant and obese, may be less motivated than those receiving an adjunctive treatment. This could lead to an overestimation of the efficacy of the association between PGR and metformin. Nevertheless, the same compliance seen with the different treatments suggests that this is not a major factor. The open label construction did not influence the evaluation of adverse reactions to the different treatments, making it possible to draw firm conclusions about the long-term safety and tolerability of metformin and the safety and tolerability of the combination therapy. However, future research should focus on conducting trials with sufficient power and follow-up to confirm the long-term effects of this combination. 

Another limitation of this study is the absence of a placebo group, which can be explained in retrospective design of the study. Further prospective studies should include a placebo group not on LGI diet to better evaluate the effect of the polysaccharide complex in improving the metabolism.

In conclusion, metformin shows short-term efficacy in reducing BMI and adiposity parameters, which is significant in obese children and adolescents with metabolic syndrome. However, further improvements in the long term appear more problematic. In the same population, PGR in combination with metformin seems to potentiate the weight reduction and improve glucose-insulin metabolism and adiposity parameters, significantly reducing the number of patients with MetS. Additional studies that include larger sample sizes and longer longitudinal follow-up times should be performed in order to confirm our results. 

## Figures and Tables

**Table 1:** Anthropometric, dietary, clinical and biochemical features before and after metformin treatment and in control group.

Features	T0 (Patients)	T0 (Controls)	T1 (Patients)	T1 (Controls)
Age, years	12.6 (8.1–14.3)	12.4 (8.2–14.5)	13.7 (9.1–15.4)	13.6 (9.2–15.5)
Tanner stage				
Prepubertal/pubertal, *n* (%)	66/63 (51.2/48.8)	27/24 (52.9/47.1)	56/73 (43.4/56.6)	22/29 (43.1/56.9)
Height, SDS	0.55 ± 0.96	0.53 ± 0.91	0.50 ± 0.93	0.56 ± 0.88
BMI, SDS	2.44 ± 0.25	2.42 ± 0.26	2.18 ± 0.21 ***	2.31 ± 0.24 *^,††^
Waist, SDS	3.14 ± 0.63	3.12 ± 0.66	2.78 ± 0.52 ***	2.99 ± 0.61 ^†^
Hip SDS	4.08 ± 0.72	4.10 ± 0.70	3.73 ± 0.65 ***	4.01 ± 0.71 ^†^
WHR	0.88 ± 0.05	0.89 ± 0.06	0.87 ± 0.02 *	0.88 ± 0.05
Acanthosis nigricans, %	76 (58.9%)	27 (52.9%)	53 (41.1%) **	28 (54.9%) ^††^
Glycemic Index	59.3 ±15.5	58.9 ±16.2	47.6 ± 7.3 ***	47.7 ± 7.2 ***
Energy intake, kcal	2,206 ± 458	2,181 ± 443	2,008 ± 364 **	1,975 ± 386 *
Fiber consumption, g	16.5 ± 7.8	16.0 ± 7.2	20.8 ± 9.4 ***	21.5 ± 9.6 **
Fat consumption, g	77.7 ± 20.2	74.9 ± 19.7	62.8 ± 20.3 ***	61.7 ± 23.4 ***
Carbohydrate consumption, g	299.0 ± 64.4	297.0 ± 69.1	286.0 ± 75.9 ***	286.5 ± 70.4 **
Protein consumption, g	94.6 ± 19.9	96.5 ± 22.3	89.8 ± 20.7	91.4 ± 23.1
HOMA-IR	7.42 ± 1.94	6.99 ± 1.81	6.11 ± 1.23 ***	6.23 ± 1.94 *
IS_OGTT_, SDS	1.22 ± 0.29	1.31 ± 0.27	1.51 ± 0.22 ***	1.42 ± 0.24 *^,†^
Insulinogenic index	3.78 ± 2.81	3.63 ± 2.70	2.95 ± 1.96 *	3.51 ± 2.04
Disposition index	4.73 ± 3.84	4.12 ± 3.71	4.53 ± 3.38	4.43 ± 3.74
Glucose post-OGTT (120 min), mg/dL	129.85 ± 28.27	132.62 ± 26.94	115.57 ± 24.16 ***	127.89 ± 23.77
HbA1C, %	6.29 ± 0.31	6.19 ± 0.32	6.01 ± 0.35 ***	6.03 ± 0.28 *
Systolic BP, SDS	1.53 ± 0.92	1.56 ± 0.99	1.45 ± 0.90	1.52 ± 0.90
Diastolic BP, SDS	1.56 ± 0.78	1.61 ± 0.84	1.35 ± 0.83 *	1.55 ± 0.73
Glucose metabolism abnormalities, n (%)				
IFG	49 (38.0%)	16 (31.4%)	28 (21.7%) ***	15 (29.4%)
IGT	31 (24.3%)	12 (23.5%)	21 (16.3%) *	11 (21.6%)
T2DM	6 (4.6%)	2 (3.9%)	5 (3.9%)	2 (3.9%)
Triglyceride, mmol/L	1.77 ± 0.31	1.88 ± 0.40	1.82 ± 0.33	1.75 ± 0.36
Total cholesterol, mmol/L	5.82 ± 0.61	5.74 ± 0.73	5.71 ± 0.62	5.56 ± 0.64
HDL-cholesterol, mmol/L	0.81 ± 0.14	0.83 ± 0.13	0.88 ± 0.14 ***	0.85 ± 0.15
LDL-cholesterol, mmol/L	4.20 ± 0.65	4.04 ± 0.59	3.98 ± 0.68 *	3.90 ± 0.57
ALT, U/L	57.23 ± 20.89	53.67 ± 18.91	52.32 ± 20.24	51.45 ± 17.76
AST, U/L	59.66 ± 27.53	55.21 ± 24.90	53.76 ± 23.19	53.45 ± 22.84

SDS, standard deviation score; BMI, body mass index; WHR, waist-hip ratio; HOMA-IR, homeostasis model of assessment for insulin-resistance; IS_OGTT_, Matsuda index; BP, blood pressure; IFG, impaired fasting glucose; IGT, impaired glucose tolerance; T2DM, type 2 diabetes mellitus. T0 patients vs. T1 patients: * *p* < 0.05; *** *p* < 0.0001. T0 controls vs. T1 controls: * *p* < 0.05; *** *p* < 0.0001. T1 patients vs. T1 controls: ^†^ *p* < 0.05; ^††^ *p* < 0.005.

**Table 2:** Anthropometric, clinical and biochemical differences during treatment with metformin or metformin plus Policaptil gel retard and in control group.

Features	T1			T2		
	Group A (Metformin + PGR)	Group B (Metformin Monotherapy)	Control Group (LGI Only)	Group A (Metformin + PGR)	Group B (Metformin Monotherapy)	Control Group (LGI Only)
Age, yrs	13.7 (9.1–15.3)	13.8 (9.2–15.4)	13.6 (9.2–15.5)	14.8 (10.2–16.3)	14.8 (10.1–16.5)	14.6 (10.2–16.5)
Tanner stage						
Prepubertal/pubertal, *N* (%)	31/40 (43.7/56.3)	25/33 (43.1/56.9)	22/29 (43.1/56.9)	26/45 (36.6/63.4%)	17/41 (29.3/70.7%)	17/34 (33.3/66.7)
Height, SDS	0.48 ± 0.88	0.52 ± 0.97	0.56 ± 0.88	0.48 ± 0.91	0.51 ± 0.96	0.54 ± 0.91
BMI, SDS	2.22 ± 0.20 ^†^	2.20 ± 0.22^†^	2.31 ± 0.24	1.92 ± 0.17 ***^,†††^	2.14 ± 0.20 ^††,‡‡‡^	2.28 ± 0.26
Waist, SDS	2.75 ± 0.58 ^†^	2.82 ± 0.45	2.99 ± 0.61	2.42 ± 0.43 **^,†††^	2.75 ± 0.43 ^†,‡‡‡^	2.96 ± 0.57
Hip SDS	3.78 ± 0.69	3.68 ± 0.62 ^†^	4.01 ± 0.71	3.30 ± 0.41 ***^,†††^	3.47 ± 0.57 ^†^	3.79 ± 0.79
WHR	0.87 ± 0.02	0.87 ± 0.03	0.88 ± 0.05	0.87 ± 0.02	0.87 ± 0.03	0.88 ± 0.03
Acanthosis nigricans, %	28 (39.4%) ^††^	25 (43.1%) ^†^	28 (54.9%)	20 (15.5%) ***^,†††^	21 (36.2%) ^††,‡‡‡^	27 (52.9%)
Glycemic Index	47.2 ± 7.1	47.8 ± 7.5	47.7 ± 7.2	46.9 ± 7.0	47.4 ± 6.8	48.3 ± 6.6
Energy intake, kcal	1989 ± 345	2,024 ± 389	1,975 ± 386	1,982 ± 357	1,993 ± 371	1,967 ± 381
Fiber consumption, g	20.1 ± 9.7	21.5 ± 9.0	21.5 ± 9.6	22.6 ± 9.0	23.6 ± 9.9	23.0 ± 9.8
Fat consumption, g	60.1 ± 19.3	64.9 ± 21.5	61.7 ± 23.4	63.1 ± 22.3	62.7 ± 24.2	61.1 ± 23.8
Carbohydrate consumption, g	271.8 ± 78.9	298.1 ± 72.3	286.5 ± 70.4	284.3 ± 78.0	288.0 ± 75.1	286.4 ± 68.9
Protein consumption, g	90.6 ± 18.6	87.7 ± 23.5	91.4 ± 23.1	89.8 ± 20.4	91.0 ± 22.9	90.9 ± 24.7
HOMA-IR	6.31 ± 1.31	5.89 ± 1.14	6.23 ± 1.94	4.12 ± 0.47 ***^,†††^	5.97 ± 1.11 ^‡‡‡^	6.56 ± 2.17
IS_OGTT_, SDS	1.47 ± 0.19	1.53 ± 0.25^†^	1.42 ± 0.24	2.27 ± 0.52 ***^,†††^	1.65 ± 0.21 *^,‡‡‡^	1.59 ± 0.38
Insulinogenic index	2.81 ± 1.69 ^†^	3.18 ± 2.29	3.51 ± 2.04	2.24 ± 1.12 *^,†††^	2.97 ± 1.98 ^‡^	3.67 ± 2.36
Disposition index	4.27 ± 2.81	4.79 ± 3.96	4.43 ± 3.74	6.78 ± 2.99 ***^,††^	4.92 ± 2.56 ^‡‡^	4.78 ± 3.34
Glucose post-OGTT (120 min), mg/dL	115.78 ± 22.69 ^†^	119.23 ± 26.57	127.89 ± 23.77	98.87 ± 24.78 ***^,††^	106.70 ± 25.65	118.31 ± 26.33
HbA1C, %	5.98 ± 0.41	6.04 ± 0.29	6.03 ± 0.28	5.71 ± 0.28 ***^,†††^	5.94 ± 0.26 ^‡‡‡^	6.03 ± 0.28
Systolic BP, SDS	1.49 ± 0.73	1.41 ± 1.08	1.52 ± 0.90	1.21 ± 0.82 *	1.33 ± 1.08	1.52 ± 0.90
Diastolic BP, SDS	1.38 ± 0.86	1.33 ± 0.82	1.55 ± 0.73	1.25 ± 0.79 ^†^	1.30 ± 0.83	1.55 ± 0.73
Glucose metabolism abnormalities, *n* (%)						
IFG	15 (21.1%)	13 (22.4%)	15 (29.4%)	5 (7.0%) ***^,†††^	9 (15.5%) ^†††^	14 (27.5%)
IGT	11 (15.5%)	10 (17.2%)	11 (21.6%)	5 (7.0%) *^,†††^	7 (12.1%)	10 (19.6%)
T2DM	3 (4.2%)	2 (3.4%)	2 (3.9%)	1 (1.4%) **^,†††^	1 (1.7%) ^††^	3 (5.9%)
Triglyceride, mmol/L	1.77 ± 0.27	1.87 ± 0.37	1.75 ± 0.36	1.58 ± 0.15 ***	1.57 ± 0.15 ***	1.68 ± 0.43
Total cholesterol, mmol/L	5.80 ± 0.58	5.63 ± 0.66	5.56 ± 0.64	5.18 ± 0.68 ***^,†^	5.52 ± 0.65 ^‡‡^	5.50 ± 0.71
HDL-cholesterol, mmol/L	0.91 ± 0.14 ^†^	0.86 ± 0.14	0.85 ± 0.15	1.06 ± 0.11 ***^,†††^	0.92 ± 0.15 ^†,‡‡‡^	0.84 ± 0.19
LDL-cholesterol, mmol/L	4.05 ± 0.65	3.82 ± 1.06	3.94 ± 0.57	3.25 ± 0.90 ***^,†††^	3.86 ± 0.68 ^‡‡‡^	3.90 ± 0.51
ALT, U/L	56.20 ± 20.45	48.50 ± 19.90	51.45 ± 17.76	40.02 ± 13.27 ***^,†††^	48.26 ± 14.98 ^‡^	52.89 ± 19.63
AST, U/L	57.85 ± 24.90	49.00 ± 22.15	53.45 ± 22.84	42.00 ± 17.10 ***^,††^	48.55 ± 16.53	55.67 ± 24.39

SDS, standard deviation score; BMI, body mass index; WHR, waist hip ratio; HOMA-IR, homeostasis model of assessment for insulin-resistance; IS_OGTT_, Matsuda index; BP, blood pressure; IFG, impaired fasting glucose; IGT, impaired glucose tolerance; T2DM, type 2 diabetes mellitus; * *p* < 0.05; ** *p* < 0.005; *** *p* < 0.0001. T1 (Group A and/or B) patients vs. T2 (Group A and/or B) patients and T1 vs. T2 controls: * *p* < 0.05; ** *p* < 0.005 *** *p* < 0.0001. T1 (Group A and/or B) patients vs. T1 controls and T2 (Group A and/or B) patients vs. T2 controls: ^†^ *p* < 0.05; ^††^ *p* < 0.005; ^†††^ *p* < 0.0001. T1 Group A patients vs. T1 group B patients and T2 group A patients vs. T2 groups B patients: ^‡^ *p* < 0.05; ^‡‡^ *p* < 0.005; ^‡‡‡^ *p* < 0.001.
